# Generation of a Golden Leaf Triploid Poplar by Repressing the Expression of *GLK* Genes

**DOI:** 10.48130/FR-2021-0003

**Published:** 2021-01-29

**Authors:** Yidi Li, Chenrui Gu, Huixin Gang, Yu Zheng, Guifeng Liu, Jing Jiang

**Affiliations:** State Key Laboratory of Tree Genetics and Breeding, Northeast Forestry University, Harbin 150040, China

**Keywords:** *GLK*, Poplar, Colorful leaf, Yellow green leaf

## Abstract

Poplar trees are excellent varieties widely used for gardening and greening. However, their single color and floating fluffy seeds are major disadvantages. Plant species or varieties with variegated leaves are desperately needed to meet various demands for gardens, urban greening and landscape decoration, as they produce rich foliage colors that are aesthetically pleasing and functional. In this study, we generated a golden leaf triploid poplar (*P.* *alba* × *P. berlinensis*) by repressing the expression of *GLK* (*Golden2-like*) genes in leaves. The triploid golden leaf poplar had reduced chlorophyll content but almost no change in the growth rate. It has great potential in landscaping once it passes safety assessments.

## INTRODUCTION

With societal and economic developments, the demand for urban garden plants with leaves of various colors has increased. Traditional plant materials used for landscaping are often monotonous and far from meeting the societal needs. Therefore, the breeding of garden plants with colorful leaves has recently gained momentum. Plants with colorful leaves can be as colorful as flowers. They can enrich urban landscapes; thus, they have become new favorites for garden greening. Among plants with colorful leaves, the yellow-leaf ornamental plants are the brightest and most eye-catching^[1]^. They also have ornamental value, making them suitable for the landscaping of gardens and courtyards.

Genetic engineering is an effective way to change the leaf color of plants. For example, the overexpression of *MYB* transcription factors can activate the expression of genes involved in anthocyanin biosynthesis, leading to the increased redness of leaves or fruit peels. Specifically, the overexpression of *Myb119* in *Poplus trichocarpa* (*P. trichocarpa*) significantly increased the anthocyanin level, resulting in red leaves^[2]^.

Golden2-like (GLK), a member of the GARP transcription factor family, widely exists in plants^[3]^. In plants, the GLKs mainly regulate chloroplast development. They are also involved in biotic and abiotic stress responses, senescence, and hormone signaling^[4]^. Studies have shown that overexpression of *AtGLK1* in *Arabidopsis thaliana* can promote the development of chloroplasts in some non-photosynthetic organs such as roots. On the contrary, the *glk1glk2* double mutant of *Arabidopsis* exhibited light green leaves, abnormal chloroplast development and decreased accumulation of chlorophyll precursors^[3,5,6,7]^. The GLK family is diverse in different species. The *Arabidopsis* *thaliana* and *Oryza sativa* genomes each contain two *GLKs*^[3]^. Recent studies have revealed that members of the GLK family may be functionally redundant while exerting different functions in different tissues^[5]^. For example, *ZmGLK1* and *ZmGLK2* in maize specifically regulate the development of sheath and mesophyll cells, respectively^[8]^. *SlGLK1* in *Solanum lycopersicum* and *CaGLK1* in *Capsicum annuum* are mainly expressed in roots, whereas *SlGLK2* and *CaGLK2* are mainly expressed in fruits^[9]^. A recent study has reported that the birch genome (*Betula platyphylla* Suk.) contains only one *GLK* gene^[10]^, and the repression of *BpGLK1* affects chloroplast development, thereby leading to yellow-green leaves. GLK family also plays an important role in response to adversity stress. Studies have shown that Arabidopsis overexpressing *AtGLK1* has enhanced resistance to the *Fusarium graminearum* and *Botrytis cinerea*^[11,12]^.

The hybrid poplar *Populus* *alba* × *P. berlinensis* is a major broadleaf tree species in Northeast China. It has been widely used for landscaping in urban roads, parks, courtyards, and residential areas because of its straight trunk, gray green bark, and beautiful appearance. In addition, *P.* *alba* × *P. berlinensis* is a triploid male polar species, which exhibits multiple advantages for landscaping^[13]^. For example, it does not generate catkins similar to other poplar species, and its male infertility can prevent gene drifting, making it an ideal material for molecular breeding. In this study, we identified *GLKs* that are highly expressed in *P.* *alba* × *P. berlinensis* leaves, and then employed gene silencing technology to generate *GLK* RNA interference (RNAi) transgenic lines with significantly reduced GLK expression levels. The transgenic lines exhibited aesthetically appealing and eye-catching yellow-green leaves. Since they are infertile triploids, they can satisfy the demands of various landscaping purposes after a safety assessment.

## RESULTS

### Identification of *GLK* genes expressed in *Populus alba* × *P. Berolinensis* leaves

The *GLK* genes are involved in chloroplast development, and the repression of *GLKs* has been reported to cause yellow leaves in multiple species^[2]^. In this study, we intended to generate a golden-yellow poplar varieties using gene silencing technology and targeted the *GLK* genes in a male triploid hybrid poplar, *P. alba* × *P. berolinensis*, for silencing. The annotated *P. trichocarpa GLKs* were used as queries to identify *PabGLKs* from the unigenes we assembled from the sequence reads from the cDNA libraries of the triploid. Finally, a total of five candidate *PabGLKs* were obtained and designated *PabGLK1* to *PabGLK5*.

**Figure 1:**
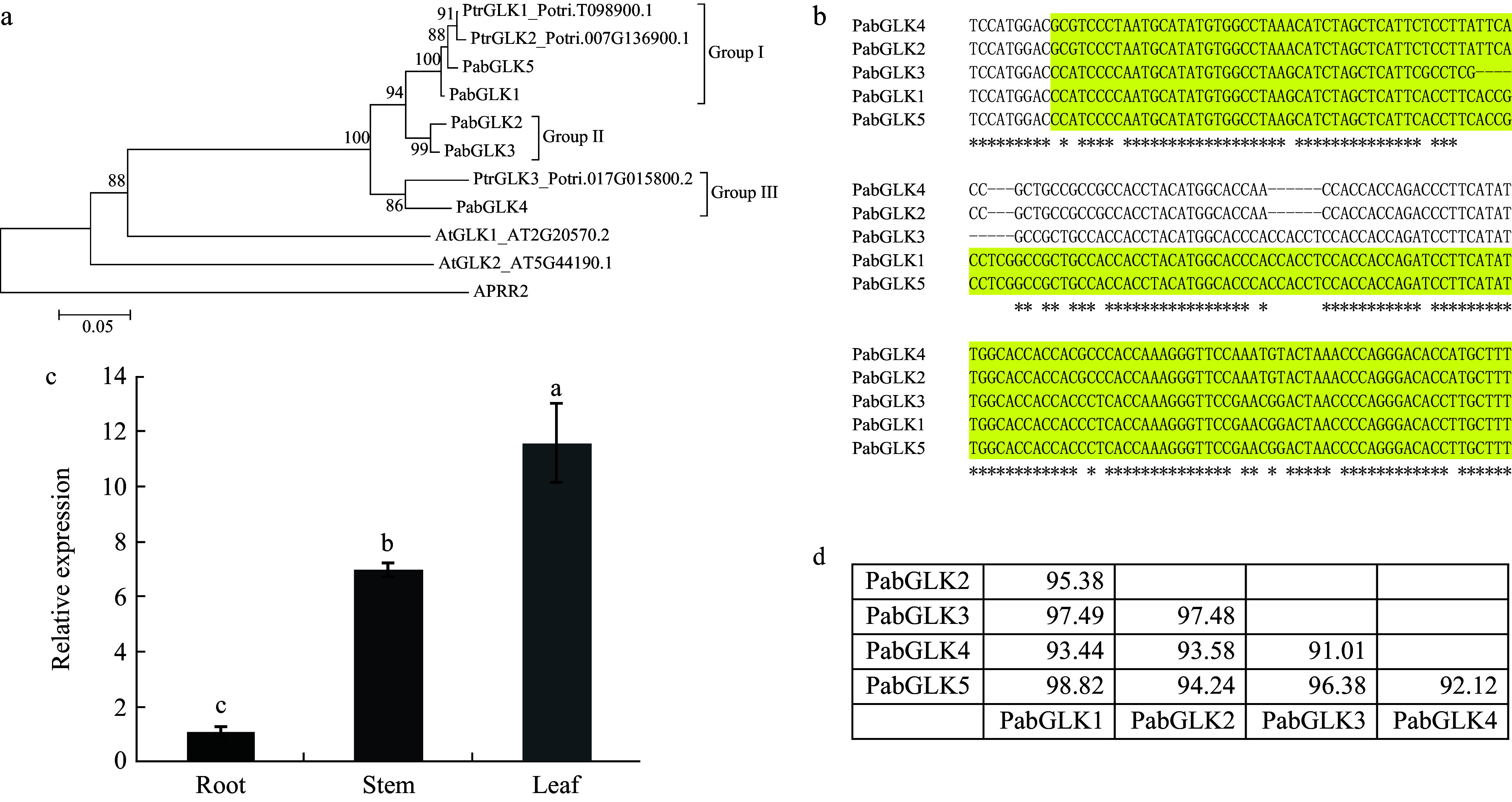
Identification of *GLKs* that are expressed in *P. alba* × *P. berolinensis*. (a): Phylogenetic relationship among *GLKs* from *P. alba* × *P. berolinensis*, *P. trichocarpa*, and *Arabidopsis*. (b): A multiple sequence alignment of the identified *PabGLKs*. The colored region was used for RNAi. (c): Tissue specific expression levels of the *PabGLKs* in *P. alba* × *P. berolinensis* quantified with qRT-PCR. 18S rRNA and α-Tubulin were used as endogenous control. (d): Nucleotide sequence similarities among differnt *PabGLKs* in *P. alba* × *P. berolinensis*.

A phylogenetic tree was constructed to reveal the genetic relationships of GLKs from *P. alba* × *P. berolinensis*, *P. trichocarpa*, and *Arabidopsis*. The results indicate that the identified *PabGLKs* could be divided into three major groups (Fig. 1a). Groups I and II each contained two *PabGLKs*; namely, *PabGLK1* and *PabGLK5* were in Group I, whereas *PabGLK2* and *PabGLK3* were in Group II. Group III had only one member, *PabGLK4*. A multiple sequence alignment of the five *PabGLKs* revealed that the nucleotide sequences of the two *PabGLKs*, *PabGLK1* and *PabGLK5,* shared the highest similarity of 98%, while *PabGLK4* and *PabGLK3*, shared the lowest similarity of 91% (Fig. 1b and d). We then measured the total expression levels of *PabGLKs* in the leaves, stems, and roots of *P. alba* × *P. berolinensis* using qPCR. The *PabGLKs* were expressed most highly in leaves, followed by stems. Roots had the lowest levels of *PabGLK* trasncripts, which were approximately 10 times lower than those of their counterparts in leaves (Fig. 1c).

### Generation of *PaGLK* repressed poplar

*GLKs* have been reported to be functionally redundant in many species. In *P. alba* × *P. berolinensis*, a total of five expressed *GLKs* were identified in leaves. Based on the results of multiple sequence alignment, we used a 200-bp region of coding region that is conserved among all *PabGLK*s for RNA silencing (Fig. 1b). An RNAi vector (p35S::*GLK*-RNAi) was designed to simultaneously silence all *PabGLK*s by targeting the selected region. The resulting RNAi construct was delivered into *P. alba* × *P. berolinensis* by *Agrobacterium*-mediated genetic transformation and three *PabGLK*s repression lines, designated RE1, RE2, and RE3, were obtained. In addition to the three *PabGLK* RNAi lines, we also generated three *PabGLK5* overexpression lines, OE1, OE2, and OE3.

**Figure 2:**
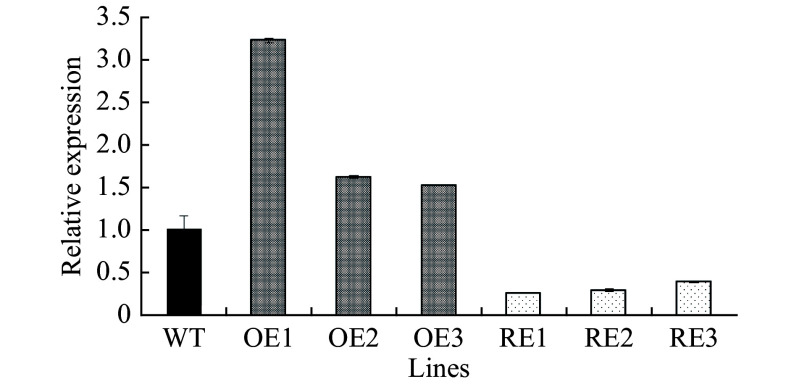
Relative *PabGLK5* transcript levels in *PabGLK5* overexpression lines and the total transcript levels of all five *PabGLKs in PabGLK* RNAi lines. OE, overexpression lines of *PabGLK5*; RE, repression lines of *PabGLKs*; WT, wildtype.

Next, the expression levels of *PabGLK5* and the total expression levels of all five *PabGLKs* in the leaves of different RE and OE lines relative to the WT were measured using qPCR. The total transcript levels of *PabGLKs* in the leaves of RE1, RE2, and RE3 lines were reduced by 74.10%, 71.10%, and 60.70%, respectively, compared with the WT. By contrast, the PabGLK5 transcript levels in the leaves of OE1, OE2, and OE3 lines increased 220.61%, 61.00%, and 51.80%, respectively (Fig. 2).

### Phenotypic analysis of *PaGLK* transgenic poplars

**Figure 3:**
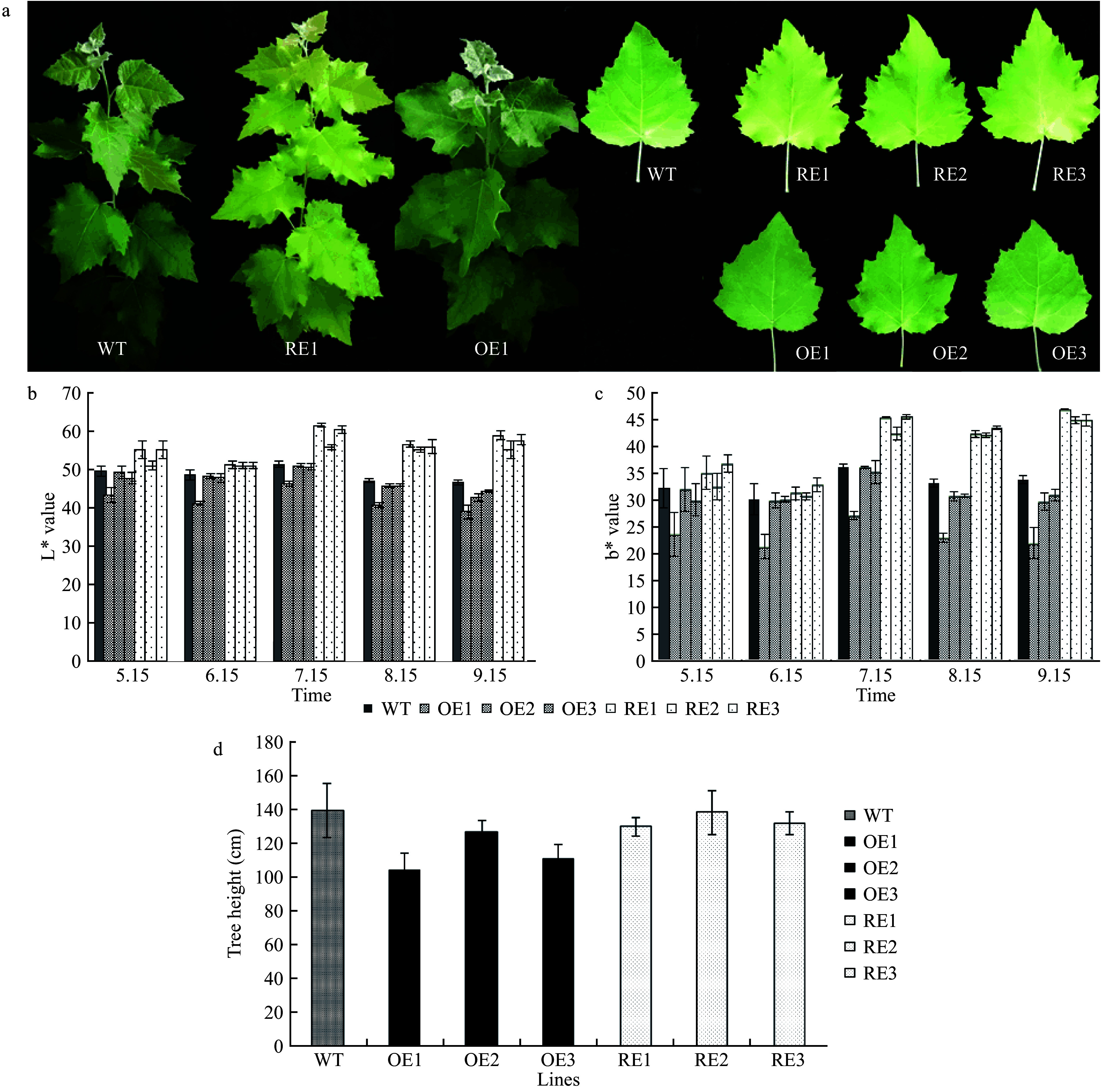
Phenotypic characterization of the *PabGLK* transgenic lines. (a): The yellow-green leaf phenotype of the *PabGLK* RNAi lines. (b) and (c): L* and b* values of the transgenic lines and WT measured by the CIELab color system. (d): Plant heights of one-year-old WT and transgenic lines. OE, overexpression lines of *PabGLK5*; RE, repression lines of *PabGLKs*; WT, wildtype.

The L* value can be used as an indicator of the brightness of leaf color in the CIELab color system, and a greater L* value indicates higher brightness. In addition, the b* value reflects the ratio of yellow/blue, and a greater b* value reveals increased yellow or decreased blue color. A colorimeter detected significantly reduced L* and b* values for *PabGLK5* OE lines or showed no significant difference compared with those of different growing periods. By contrast, the L* and b* values for *PabGLK* RNAi lines were significantly higher than those of the WT. In particular, on July 15, the mean values of L* and b* for the three *PabGLKs* RNAi lines were 15.40% and 22.40% higher than those for the WT, respectively (Fig. 3a−c), suggesting that repressing the expression of *PabGLKs* not only turned *P. alba* × *P. berolinensis* leaves yellow but also enhanced the brightness, as measured by the CIELAB system.

The average heights of one-year-old *PabGLK* RNAi lines were comparable to that of the WT, whereas those of *PabGLK5* overexpression lines (G line) were 18.23% lower than the WT (*P* < 0.05, Fig. 3d).

### Determination of pigment contents and photosynthetic and fluorescence parameters

**Figure 4:**
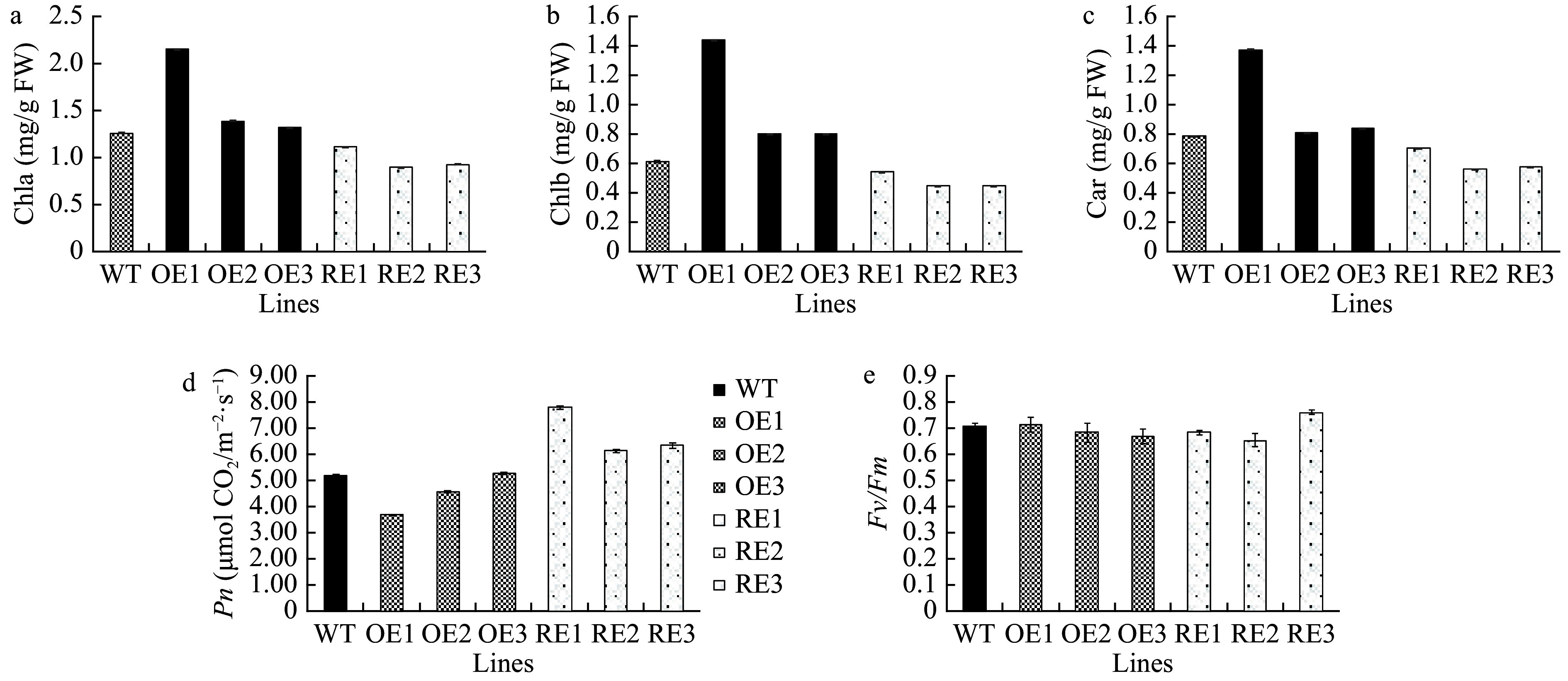
Pigment contents and photosynthetic and fluorescence parameters of the WT and transgenic lines. OE, overexpression lines of *PabGLK5*; RE, repression lines of *PabGLKs*; WT, wildtype. (a), (b) and (c) show the contents of chlorophyll a, chlorophyll b, and carotenoid in the WT and transgenic lines, respectively. (d) and (e) show photosynthetic parameters *Pn* and *Fv*/*Fm*, *respectively*.

The contents of chlorophyll a, chlorophyll b, and carotenoids significantly increased by 28.5%, 65.9%, and 28.3%, respectively, in *PaGLK5* overexpression lines compared with the WT. By contrast, the contents of chlorophyll a, chlorophyll b, and carotenoids in the *PaGLK* RNAi lines significantly decreased by 22.3%, 21.9%, and 21.7%, respectively, compared with the WT (Fig. 4a−c). The net photosynthesis (*Pn*) of *PaGLK5* overexpression lines significantly decreased by 20.83% compared with the WT (except for OE3), whereas that of the *PaGLK* RNAi lines were significantly higher than the WT by 29.74% (*P* < 0.05, Fig. 4d). In contrast to the changes in pigment contents and *Pn*, none of the transgenic lines showed changes in *Fv/Fm* (Fig. 4e). Taken together, these results indicate that despite the reduction in the contents of photosynthetic pigments, the photosynthetic efficiency and the maximum photochemical quantum efficiency of PS II were not affected in *PaGLK* RNAi lines.

### Expression profile of photosynthesis-related genes

**Figure 5:**
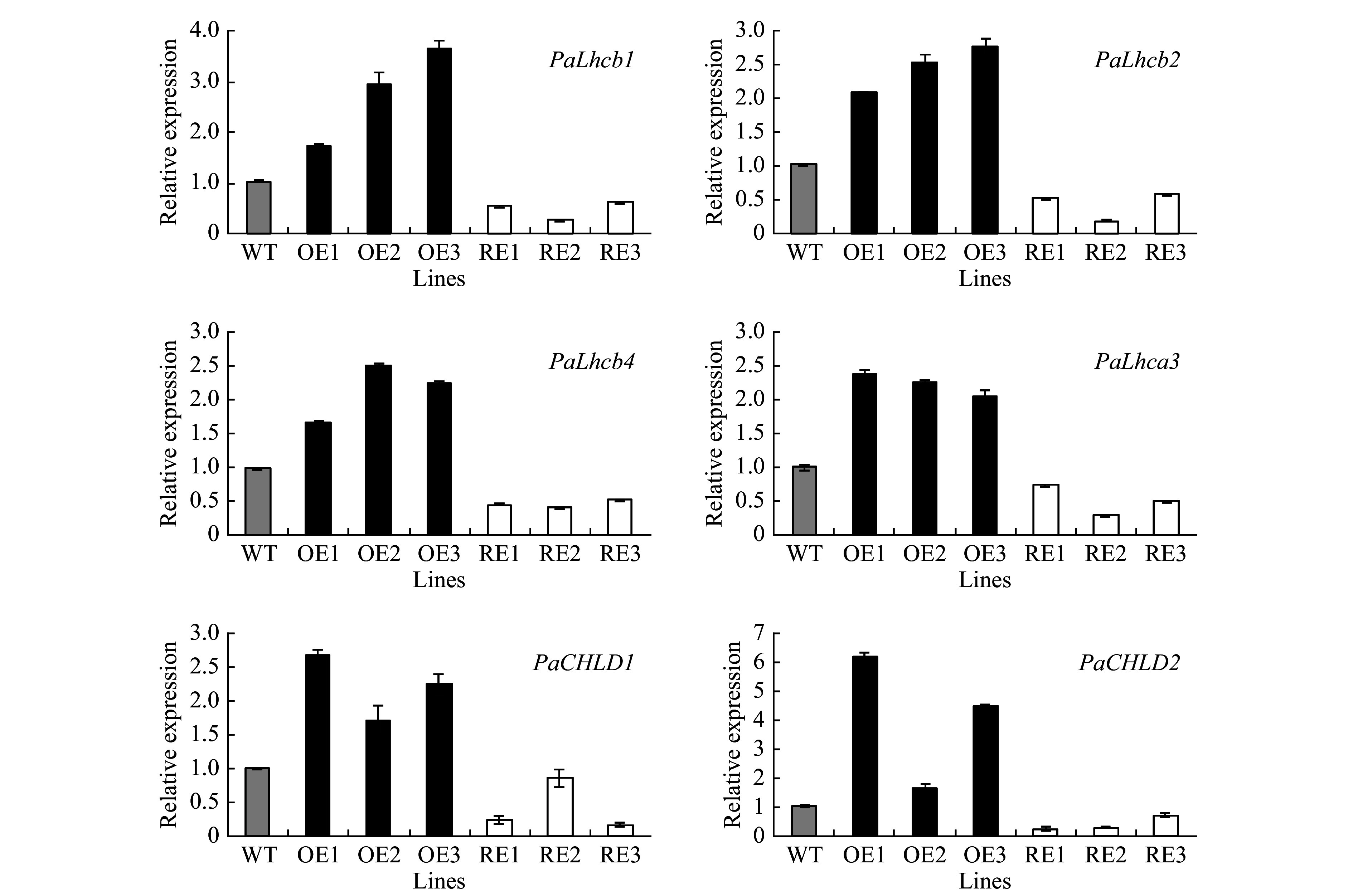
Expression profiles of photosynthesis-related genes in transgenic poplar. OE, overexpression lines of *PabGLK5*; RE, repression lines of *PabGLKs*; WT, wildtype.

The expression profiles of genes encoding the light-harvesting complex components (*PabLhcb1*, *PabLhcb2*, *PabLhcb4*, and *PabLhca3*) and genes involved in chlorophyll synthesis (*PabCHLD1*, *PabCHLD2*) in the *PabGLK* RNAi lines and *PabGLK5* overexpression lines were investigated using qPCR. The results showed that all the tested genes were significantly down-regulated in *PabGLK* RNAi lines but up-regulated in *PabGLK5* overexpression lines compared with the WT (Fig. 5).

## DISCUSSION

Generation of the first transgenic plant in 1983 has brought about broad prospects for plant genetic engineering^[14]^. However, the application of transgenic plants has become increasingly controversial, especially with the drift of transgenic pollen, which is a major aspect for the ecological safety of transgenic plants^[15]^. The plant material used in this study is a triploid male clone selected from the cross between *P. alba* Linn and *P. berolinensis* Dipp. It has a straight trunk, gray bark, beautiful tree posture, and displays male infertility. The unique characteristics of this hybrid poplar make it an ideal material for urban gardening^[16]^. Gene drifting to non-transgenic and wild varieties can be avoided in transgenic poplar generated using this variety because of its male infertility. Thus, the golden leaves of the poplar generated here by genetic engineering are ideal for urban landscaping.

T-DNA insertion sites in transgenic plants are usually random, sometimes resulting in differences in phenotypes between transgenic lines^[17]^. Therefore, the generated transgenic lines often need to be selected for several generations, and only those that exhibit the desired phenotypes can be applied. Here, the plant height and growth rate of the *PabGLK* RNAi lines were not affected; the average height of one-year-old *PabGLK* RNAi plants was 132.58 cm, comparable to that of the WT. By contrast, the plant heights of one-year-old *PabGLK5* overexpression lines were significantly lower than that of the WT, which may have been a result of the increased contents of photosynthetic pigments in the OE lines. The increased amount of pigments in the leaves of *PabGLK5* OE lines may have absorbed excessive light energy, leading to light inhibition.

Studies of *Arabidopsis* and *Betula platyphylla* have shown that *GLKs* mainly affects the synthesis of photosynthetic pigments and the development of chloroplasts by regulating the expression of genes encoding light harvesting antenna proteins and photosystem complex proteins, as well as those involved in chlorophyll synthesis^[10,3]^. Grana are the thylakoid structures accumulated in the chloroplasts of terrestrial plants. Photosystem II and light-harvesting complex II (LHCII) are mainly concentrated in the stacked grana. Photosystem I and light-harvesting complex I (LHCI) are mainly concentrated in the stroma lamella. If the protein content of the grana lamella and stromal lamella is reduced in the chloroplasts, it affects the development of the chloroplasts^[18]^. The green-yellow leaf phenotypes caused by *GLK* repression are known to perform stably in the field in many species. Therefore, the *GLK* genes are top choice candidate genes for the generation of species with different leaf colors in tree breeding.

## MATERIALS AND METHODS

### Plant materials

In this study, the 2 month-old tissue-cultured plantlets of *P. alba* × *P. berlinensis* were used for RNA extraction for *GLK* cloning and gene transformation. The plantlets were vegetatively cultured from apical meristems and stem segments excised from P. alba × P. berlinensis triplod plants that had been maintained in tissue culture room for multiple generations. The medium used was the half-strength Murashige and Skoog (1/2 MS) (Phytotech) containing 20 g/L sucrose with pH 5.8 before adding 3 g l-1 Gelrite (Duchefa). The karyotype analysis of this *P. alba* × *P. berlinensis* triploid, which had been planted in the Northeast region of China for many years, was conducted earlier^[19]^.

### Identification of *PabGLKs*

Total RNA was extracted from the leaves of the *P. alba* × *P. berlinensis* triploid plantlets using the CTAB method^[20]^. An RNA sequencing library was constructed using the NEBNext RNA Library Prep Kit for Illumina (New England Biolabs, Ipswich, MA, USA). The cDNA library was then sequenced on an Illumina sequencing platform (HiSeq™ 4 000). The resulting reads were qualified using FastQC and assembled into contigs using Trinity^[21]^ with default parameters. The assembled contigs were BLASTed^[22]^ against the transcripts of *P. trichocarpa*^[23]^ to identify expressed *GLKs* in the leaves of the triploid poplar. A multiple sequence alignment of *GLKs* from *P. trichocarpa*, *P.* *alba* × *P. berlinensis*, and *Arabidopsis* was performed using MUSCLE program^[24]^ and a neighbor-joining phelogenetic tree was constructed using a bootstrap of 1 000 replicates in MEGA6^[25]^.

### Tissue specific expression profiles of *PabGLKs*

Total RNA was extracted from the roots, stems, and leaves of two-month old *P. alba* × *P. berlinensis* triploid plantlets using the CTAB method^[26,27]^, and cDNA was synthesized using the ReverTra Ace qPCR RT Master Mix with gDNA Remover (Toyobo, Osaka, Japan). qPCR was performed using SYBR Green Real-Time PCR Master Mix Plus (Toyobo) following the manufacturer’s recommendations on an ABI PRISM 7 500 Real-Time PCR system (Applied Biosystems, Foster City, CA, USA). The relative transcript levels of the target genes were normalized to those of *18S* *rRNA* and α-*Tubulin*, by the 2^−ΔΔCT^ method^[28]^.

### Generation of transgenic poplar

To repress the expression of *PabGLKs,* a 200-bp fragment of *PabGLK5* was cloned from leaves of *P. alba* × *P. berlinensis* and inserted into the RNAi vector pFGC5941 in forward and reverse directions. Primers used for RNAi vector construction were listed in Table 1. To overexpress *PabGLK5*, the full-length cDNA sequence of *PabGLK5* was cloned into the plant expression vector pCAMBIA1300. The resulting RNAi and overexpression constructs were introduced into the *P.* *alba* × *P. berlinensis* genome through *Agrobacterium*-mediated transformation to generate *PabGLK* repression and *PabGLK5* overexpressing lines. Genetic transformation was performed as described previously^[22]^.

**Table 1:** Primers used for RNAi vector construction.

Primers	Sequences (5'-3')
*PaGLK*-RNAi-Cis-*Nco*I	CATGCCATGGCCATCCCCAATGCATATGTG
*PaGLK*-RNAi-Cis-*Asc*I	TTGGCGCGCCGCAGGAAATCTAGTTGCCAGT
*PaGLK*-RNAi-Anti- *Xba*I	GCTCTAGACCATCCCCAATGCATATGTG
*PaGLK*-RNAi-Anti-*BamH*I	CGCGGATCCGCAGGAAATCTAGTTGCCAGT

### Determination of pigment contents and photosynthetic parameters

Chl and carotenoids (Car) were extracted with 80% acetone at 4 °C for 24 h in the dark and measured at 470, 645, and 663 nm with a UV-Vis spectrophotometer (TU-1901, PERSEE, Suzhou, China). Three individual plants were sampled for each line, and three leaves per plant were measured. The photosynthetic and kinetic parameters of Chl fluorescence were measured for the mature leaves of the WT and transgenic lines using a portable photosynthesis system (Li-6400, LI-COR, Nebraska USA).

### Measurement of leaf colors of transgenic lines

A spectrocolorimeter (KONICA MINOLTA CR-400) was used to measure the leaf colors of the transgenic lines. For each line, 30 different individual plants were measured, with one leaf per plant. The measurement starts on 15 May and ends on 15 September, and the measurement was performed once in every 30 days. The measurement results were analyzed by the CIELab color system, where L* represents the brightness (Lumination) value between black and white, with a value in the range of 0−100, while b* represents blue-yellow opponents with positive numbers toward yellow and negative numbers toward blue (yellow (+b) blue (−b)).

### Statistical analysis

SPSS statistical analysis software and Excel software were used for variance analysis and multiple comparisons.
